# Characterization of Flavin-Containing Opine Dehydrogenase from Bacteria

**DOI:** 10.1371/journal.pone.0138434

**Published:** 2015-09-18

**Authors:** Seiya Watanabe, Rui Sueda, Fumiyasu Fukumori, Yasuo Watanabe

**Affiliations:** 1 Faculty of Agriculture, Ehime University, Matsuyama, Ehime, Japan; 2 Center for Marine Environmental Studies, Ehime University, Matsuyama, Ehime, Japan; 3 Faculty of Food and Nutritional Sciences, Toyo University, Itakura-machi, Gunma, Japan; Centre National de la Recherche Scientifique, Aix-Marseille Université, FRANCE

## Abstract

Opines, in particular nopaline and octopine, are specific compounds found in crown gall tumor tissues induced by infections with *Agrobacterium* species, and are synthesized by well-studied NAD(P)H-dependent dehydrogenases (synthases), which catalyze the reductive condensation of α-ketoglutarate or pyruvate with L-arginine. The corresponding genes are transferred into plant cells via a tumor-inducing (Ti) plasmid. In addition to the reverse oxidative reaction(s), the genes *noxB-noxA* and *ooxB*-*ooxA* are considered to be involved in opine catabolism as (membrane-associated) oxidases; however, their properties have not yet been elucidated in detail due to the difficulties associated with purification (and preservation). We herein successfully expressed Nox/Oox-like genes from *Pseudomonas putida* in *P*. *putida* cells. The purified protein consisted of different α-, β-, and γ-subunits encoded by the *OdhA*, *OdhB*, and *OdhC* genes, which were arranged in tandem on the chromosome (*OdhB-C-A*), and exhibited dehydrogenase (but not oxidase) activity toward nopaline in the presence of artificial electron acceptors such as 2,6-dichloroindophenol. The enzyme contained FAD, FMN, and [2Fe-2S]-iron sulfur as prosthetic groups. On the other hand, the gene cluster from *Bradyrhizobium japonicum* consisted of *OdhB*_*1*_ *-C-A-B*_*2*_, from which two proteins, OdhAB_1_C and OdhAB_2_C, appeared through the assembly of each β-subunit together with common α- and γ-subunits. A poor phylogenetic relationship was detected between OdhB_1_ and OdhB_2_ in spite of them both functioning as octopine dehydrogenases, which provided clear evidence for the acquisition of novel functions by “subunit-exchange”. To the best of our knowledge, this is the first study to have examined flavin-containing opine dehydrogenase.

## Introduction

Opines are unique compounds found in crown gall tumor tissues induced by infections with pathogenic, soil-inhabiting *Agrobacterium* species including *Agrobacterium tumefaciens*, *Agrobacterium rhizogenes*, and *Agrobacterium vitis*. During infections, they transfer the so-called “T-DNA fragment”, which contains several genes for the synthesis of opines, into plant cells using a tumor-inducing (Ti) plasmid. Since genes for the degradation of opines are also contained in another non-transferred Ti plasmid, the (constitutive) production of opines by crown gall tumor tissues provides the inciting *Agrobacterium* strain with a selective growth substrate that favors its propagation (i.e., the “opine concept” [1]). Opines have been structurally classified into several groups, among which two groups have a common secondary amine dicarboxylic acid structure. One group has been categorized as the *N* ^2^-(l-D-carboxyethyl) derivatives of L-arginine (octopine), L-ornithine (octopinic acid), L-lysine (lysopine), L-histidine (histopine), L-methionine (methiopine), and L-phenylalanine (phenylalaninopine) [2, 3, 4]. The second group has been categorized as the *N* ^2^-(1,3-D-dicarboxypropyl) derivatives of L-arginine (nopaline), L-ornithine (nopalinic acid (ornaline)), L-leucine (leucinopine), and L-asparagine (succinamopine) [5, 6, 7].

**Fig 1:**
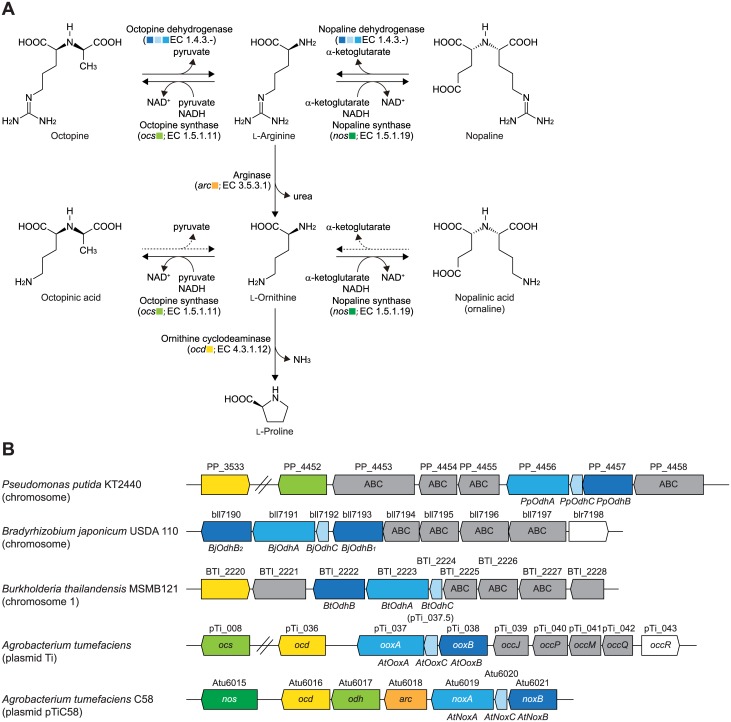
Metabolic network of opines. (A) Metabolic networks of nopaline, octopine, nopalinic acid, and octopinic acid. NOS and OCS also synthesize nopalinic acid and octopinic acid, respectively [5, 11]. The inhibition experiment performed in this study revealed that nopalinic acid and octopinic acid may be active substrates for flavin-containing dehydrogenases for nopaline and octopine, respectively (dashed arrow). (B) Schematic gene clusters related to the metabolism of opines by bacteria. Homologous genes are indicated in the same color and correspond to Fig 1A. Gray genes are putative opine transporters.

Of these, octopine [*N* ^2^-(l-D-carboxyethyl)-L-arginine] and nopaline [*N* ^2^-(1,3-D-dicarboxypropyl)-L-arginine] are synthesized by NAD(P)H-dependent soluble dehydrogenases that catalyze the reductive condensation of pyruvate (for octopine) or α-ketoglutarate (for nopaline) with L-arginine (Fig 1A). Although these reactions may be reversible *in vitro*, the frequent use of the term “synthase” rather than “dehydrogenase” has emphasized the importance of “biosynthesis”, but not “degradation”, and distinguishes them from the mollusk octopine dehydrogenase [8]: octopine synthase (EC 1.5.1.11; OCS) and nopaline synthase (EC 1.5.1.19; NOS). OCS and NOS from *A*. *tumefaciens*, encoded by the *ocs* (pTi_008) and *nos* genes (Atu6015), respectively, belong to the same protein superfamily (pfam02317) [9, 10], and a recent study reported that OCS utilized not only L-arginine, but also other amino acids, to yield the corresponding members of the octopine family [11]. Furthermore, NOS has been suggested to synthesize nopalinic acid [5].

The octopine catabolic (*occ*) operons of octopine-type Ti plasmids consist of at least 15 genes that include ABC-type (opine) permease (encoded by *occQMPJ*), ornithine cyclodeaminase (*ocd*), and methionine synthase (*msh*) [11] (Fig 1B). On the other hand, genes including *nos*, related to nopaline metabolism, are known to be encoded at the left part of the nopaline catabolic (*noc*) region in the pTiC58 plasmid [12]. Furthermore, both the *occ* operon and *noc* region also contain several genes suspected of being involved in opine metabolism such as *ooxA*, *ooxB*, *noxA*, and *noxB*, which were the focus of this study. In both opines, the first step of degradation is the reverse of biosynthesis, i.e. oxidative cleavage to L-arginine and pyruvate or α-ketoglutarate (L-arginine may subsequently be metabolized to L-proline via L-ornithine through two sequential enzyme reactions by arginase (EC 3.5.3.1) and ornithine cyclodeaminase (EC 4.3.1.12)) (Fig 1A).

More than twenty years ago, Zanker *et al*. [13] reported that when *noxB*-*noxA* and *ooxB*-*ooxA* within the *occ* operon and *noc* region of Ti plasmids were transcriptionally fused with the vector promoter and expressed in *Escherichia coli* cells, cofactor-independent oxidase activities for nopaline and octopine were observed in the membrane fraction. However, these activities were directly assayed using the *E*. *coli* lysate with lysozyme (without centrifugation) because of serious problems with the resuspension of the membrane. Although 38 and 36% sequence identities have been reported between NoxB and OoxB, and NoxA and OoxA, respectively, no protein with extensive overall similarity to either of them was found in the database. Until now, there has been no clear evidence to show that these genes (proteins) function as opine oxidase. We recently characterized the bacterial pathway of *trans*-4-hydroxy-L-proline metabolism, in which flavin-containing D-hydroxyproline dehydrogenase (EC 1.4.99.−; D-HypDH) catalyzes the second reaction step. In case of *Pseudomonas aeruginosa* [14] and *Azospirillum brasilense* [15], the enzyme has a heterododecameric structure that consists of three different subunits (α_4_β_4_γ_4_), and the genes encoding the β-, γ-, and α-subunits are arranged in tandem on the bacterial genome. Since the β- and α-subunits show significant sequence similarities to NoxB and OoxB, and NoxA and OoxA, respectively, it currently remains unclear whether (putative) Nox and/or Oox protein(s) are actually encoded by only two genes.

Bacteria capable of growing on opines as the sole carbon/nitrogen source have been isolated not only from tumors, but also soil and the rhizosphere, and are not limited to *Agrobacterium* species [16, 17]. Only one study has examined catabolism by these bacteria at the molecular level. Asano *et al*. [18, 19] reported that *Arthrobacter* sp. strain 1C, isolated from soil, grew on *N* ^2^-[l-D-(carboxyl)ethyl-L-phenylalanine] as the sole carbon source, and possessed NAD^+^-dependent dehydrogenase that catalyzed a reversible oxidization-reduction reaction of several octopine-type opines including *N* ^2^-[l-D-(carboxyl)ethyl-L-methionine] and *N* ^2^-[l-D-(carboxyl)ethyl-L-phenylalanine], which were later discovered in crown gall tumor tissues as methiopine [3] and phenylalaninopine [4]. The enzyme is sequentially homologous to OCS and NOS, while the corresponding gene is encoded on the chromosome (but not the plasmid). Thus, the physiological roles of opines for non-*Agrobacterium* species in nature have not yet been elucidated in detail.

In the present study, we focused on Nox/Oox-like protein(s) from *Pseudomonas putida* KT2440 [20], which is frequently isolated from soil. The first biochemical characterization of the recombinant protein(s), successfully expressed in *P*. *putida* cells, revealed that the protein consisted of three different subunits, and functioned as a novel type of flavin-containing opine (nopaline) dehydrogenase, which differed from known NAD(P)^+^-dependent enzymes. On the other hand, the gene cluster of *Bradyrhizobium japonicum* USDA110, a nitrogen-fixing symbiotic bacterium, consisted of “four” genes, in which two different genes that corresponded to catalytic subunit were contained. Each catalytic subunit, not related phylogenetically, assembled together with common regulatory subunits in order to function as octopine dehydrogenase, thereby providing evidence for the acquisition of novel functions by “subunit-exchange”.

## Materials and Methods

### Materials

*P*. *putida* KT2440 and *B*. *japonicum* USDA110 were purchased from the National Institute of Technology and Evaluation (Chiba, Japan). Nopaline and octopine were from Toronto Research Chemicals Inc. (Ontario, Canada).

### General procedures

Basic recombinant DNA techniques were performed as described by Sambrook *et al*. [21]. Bacterial genomic DNA was prepared using a DNeasy Tissue Kit (Qiagen). PCR was carried out using GeneAmp PCR System 2700 (Applied Biosystems) for 30 cycles in 50 μl of reaction mixture containing 1 U of KOD FX DNA polymerase (TOYOBO), appropriate primers (15 pmol), and template DNA under the following conditions: denaturation at 98°C for 10 s, annealing at 50°C for 30 s, and extension at 68°C for time periods calculated at an extension rate of 1 kbp∙min^-1^. DNA sequencing was carried out using the BigDye Cycle Sequencing Kit ver.3.1 (Applied Biosystems) and appropriate primers with the Genetic Analyzer 3130 (Applied Biosystems). High-pressure liquid chromatography (HPLC) was performed using an Agilent 1120 Compact LC system (TOSOH). Protein concentrations were determined by the method of Lowry *et al*. [22] with bovine serum albumin as the standard. SDS-PAGE was performed as described by Laemmli [23]. In this study, the prefixes Pp (*P*. *putida*), At (*A*. *tumefaciens*), Bj (*B*. *japonicum*), and Bt (*Burkholderia thailandensis*) were added to gene symbols or protein designations where necessary for clarity.

### Plasmid construction for expression of *PpOdhABC* genes in *P*. *putida* cells

**Fig 2:**
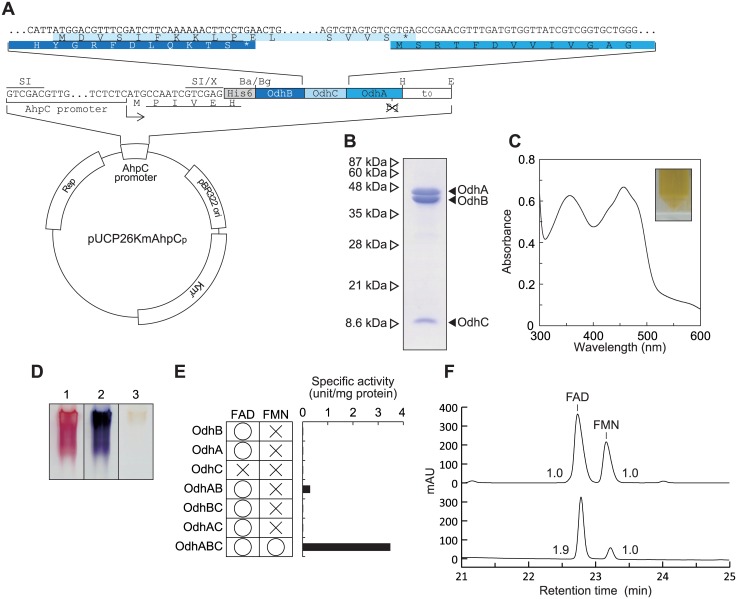
Characterization of opine dehydrogenase from *P*. *putida*. (A) Plasmid construction for the expression of *PpOdhABC* genes in *P*. *putida* cells. The inset shows the nucleotide sequences of the *PpOdhB*, *PpOdhC*, and *PpOdhA* genes at the intergenic regions along with the corresponding deduced amino acid sequences. N-terminal amino acid sequences, determined using the purified enzyme (Fig 2B), are indicated by underlining. SI, X, Ba, Bg, H, and E indicate SalI, XhoI, BamHI, BglI, HindIII, and EcoRI restriction enzyme sites, respectively. The BglI site within the *PpOdhA* gene was removed without changing the amino acid sequence. (B) An SDS-PAGE analysis of purified recombinant PpOdhABC (20 μg in 15% (w/v) gel). (C) Absorption spectra using 10 mg/ml enzyme solution (inset). (D) A zymogram staining analysis used nopaline (lanes 1 and 2) and octopine (lane 3) as substrates together with the PMS/INT (lanes 1 and 3) and PMS/NBT assay systems (lane 2). Purified enzymes of 50 μg were applied. (E) Functional characterization of α-, β-, and γ-subunits, corresponding to PpOdhA, PpOdhB, and PpOdhC, respectively. All (His)_6_-tagged proteins were expressed in *E*. *coli* cells, and purified using a Ni-NTA column. Prosthetic groups were analyzed by HPLC (Fig 2F). (F) Elution profiles of the standard mixture of FAD and FMN (upper) and extract of PpOdhABC (lower). Numbers with peaks are the molar ratio of FAD:FMN.

The primer sequences used in this study are shown in S1 Table. Synthetic oligonucleotide primers involving mutations, with no change in the amino acid sequence, were designed in order to remove the internal BglII site in *PpOdhB-OdhC-OdhA* (GenBank^TM^ accession numbers PP_4457 and PP_4456) (Fig 1B). In the first round, two reactions, I and II, were performed with the following primers and genomic DNA of *P*. *putida* as a template: I) P1 and P8 (one of the antisense primers containing the mutation); II) P7 (one of the sense primers containing the mutation) and P2. In the final amplification step, purified overlapping PCR products were used as templates, and P1 and P2 as primers. The final PCR product was introduced into the BamHI-HindIII site in pQE-81L (Qiagen), a plasmid vector for conferring the N-terminal (His)_6_-tag on expressed proteins, to obtain the plasmid pQE/PpOdhABC. A DNA fragment of the (His)_6_-PpOdhABC-*t*_0_ terminator was amplified by PCR using pQE/PpOdhABC as a template, and introduced into the SalI-EcoRI sites in pUCP26KmAhpC_p_ [24] to obtain pUCP/PpOdhABC (Fig 2A).

### Plasmid construction for expression of *PpOdhABC*, *BjOdhAB*_*1*_ *C*, *BjOdhAB*_*2*_ *C*, and *BjOdhAB*_*1*_ *B*_*2*_ *C* genes in *E*. *coli* cells

The (His)_6_-tag (H_6_) or S-tag (S) were attached to the N- or C-termini of genes (proteins) using the pETDuet-1, pACYCDuet-1, or pCOLADuet-1 vector (Novagen), respectively. *PpOdhB*, *PpOdhA*, and *PpOdhC* genes were introduced into the BamHI-HindIII sites of pACYCDuet-1, EcoRI-HindIII sites of pETDuet-1, and BamHI-HindIII sites of pCOLADuet-1, to obtain pACYC/(H_6_)PpOdhB, pET/(H_6_)PpOdhA, and pCOLA/(H_6_)PpOdhC, respectively. *BjOdhB*_*1*_ (bll7193), *BjOdhB*_*2*_ (bll7190), *BjOdhA* (bll7191), and *BjOdhC* genes (bll7192) were introduced into the BamHI-HindIII sites of pACYCDuet-1 (for *BjOdhB*_*1*_ and *BjOdhB*_*2*_), NdeI-XhoI sites of pETDuet-1 (for *BjOdhA*) or pCOLADuet-1 (for *BjOdhC*), to obtain pACYC/(H_6_)BjOdhB_1_, pACYC/(H_6_)BjOdhB_2_, pET/(S)BjOdhA, and pCOLA/(S)BjOdhC, respectively. A plasmid of pACYC/(H_6_)BjOdhB_2_(S)BjOdhB_1_ was constructed by introducing the *BjOdhB*_*1*_ gene into the NdeI-XhoI sites of pACYC/(H_6_)BjOdhB_2_.

### Expression and purification of the recombinant protein

*P*. *putida* KT2442-oxyR1 [25] harboring pUCP/PpOdhABC, obtained by the heat-shock transformation method, was grown at 30°C overnight in LB medium containing 50 mg/liter kanamycin. On the other hand, *E*. *coli* strain BL21(DE3) (Novagen) harboring the pETDuet-1, pACYCDuet-1, or pCOLADuet-1 plasmid(s) was grown at 37°C to a turbidity of 0.6 at 600 nm in Super broth medium (pH 7.0, 12 g tryptone, 24 g yeast extract, 5 ml glycerol, 3.81 g KH_2_PO_4_, and 12.5 g K_2_HPO_4_ per liter) containing chloramphenicol (30 mg/liter for pACYCDuet-1), ampicillin (50 mg/liter for pETDuet-1), and/or kanamycin (50 mg/liter for pCOLADuet-1). After the addition of 1 mM isopropyl-β-D-thiogalactopyranoside (IPTG), the culture was grown for a further 18 h at 18°C to induce the expression of the (His)_6_-tagged or S-tagged protein. Grown cells were harvested by centrifugation at 30,000 × *g* for 20 min, suspended in Buffer A (50 mM sodium phosphate buffer (pH 8.0) containing 300 mM NaCl, 1% (v/v) Tween-20, and 10 mM imidazole), disrupted by sonication for 20 min at appropriate intervals on ice using Ultra Sonic Disruptor UD-211 (TOMY SEIKO Co., Ltd, Tokyo, Japan), and then centrifuged at 108,000 × *g* for 20 min at 4°C. The supernatant was loaded onto a Ni-NTA Superflow column (Qiagen) equilibrated with Buffer A linked to the BioAssist eZ system (TOSOH). The column was washed with Buffer B (50 mM sodium phosphate buffer (pH 8.0) containing 300 mM NaCl, 0.1% (v/v) Tween-20, 10% (v/v) glycerol, and 50 mM imidazole). The enzymes were then eluted with Buffer C (pH 8.0, Buffer B containing 250 mM imidazole instead of 50 mM imidazole), concentrated by ultrafiltration with Amicon^®^ Ultra-15 (Millipore), dialyzed against 50 mM Tris-HCl buffer (pH 8.0) containing 50% (v/v) glycerol, and stored at –35°C until use.

The native molecular mass of the recombinant protein was estimated by gel filtration, which was carried out using a HPLC system at a flow rate of 1 ml/min. The purified enzyme was loaded onto a TSKgel G3000SWXL column (TOSOH) equilibrated with 50 mM Tris-HCl buffer (pH 8.0). A high molecular weight gel filtration calibration kit (GE Healthcare) was used for molecular markers.

### Western blot analysis

The purified enzyme was separated by SDS-PAGE, and the proteins on the gels were transferred onto a PVDF membrane (Hybond-P; GE Healthcare). A Western blot analysis was carried out using the ECL Western Blotting Analysis System (GE Healthcare) and appropriate antibodies: for the (His)_6_-tagged protein, an RGS∙His HRP antibody (Qiagen); for the S-tagged protein, an S-protein HRP Conjugate (Novagen).

### Determination of N-terminal amino acid sequences

As described in the Results section, when the *PpOdhABC* gene cluster was expressed in *P*. *putida* cells, the purified enzyme consisted of three bands with different molecular masses in an SDS-PAGE analysis (see Fig 2B). In order to identify these proteins with molecular masses of 42, 9, and 45 kDa as PpOdhB, PpOdhC, and PpOdhA, respectively, the purified enzyme was separated by SDS-PAGE, transferred onto a PVDF membrane, and subjected to a western blot analysis. Their N-terminal amino acid sequences were determined using a Procise 492 HT protein sequencer (Applied Biosystems).

### Enzyme assay

Opine dehydrogenase activity was spectrophotometrically assayed at 30°C by monitoring the reduction rate of Cl2Ind using a Shimadzu UV-1800 spectrophotometer (Shimadzu GLC Ltd., Tokyo, Japan). The standard reaction mixture contained 0.05 mM Cl2Ind in 50 mM Tris-HCl buffer (pH 9.0) (for PpOdhABC and BjOdhAB_2_C) or 50 mM glycine-NaOH (pH 9.0) (for BjOdhAB_1_C). The reaction was started by the addition of 10 mM nopaline or octopine (100 μl) with a final reaction volume of 1 ml. The millimolar absorption coefficient (ε) for Cl2Ind was 19.1 mM^-1^·cm^-1^ at 600 nm. One unit was defined as the amount of the enzyme catalyzing the reduction of 1 μmol of Cl2Ind/min. In order to estimate the specificity of electron acceptors, *p*-iodonitrotetrazolium violet (INT) or nitroblue tetrazolium (NBT) together with phenazine methosulfate (PMS) (electron-transfer intermediate) (15.0 mM^-1^·cm^-1^ at 490 nm and ε = 36.0 mM^-1^·cm^-1^ at 530 nm, respectively), ferricyanide (ε = 1.04 mM^-1^·cm^-1^ at 405 nm), horse heart cytochrome *c* (Sigma) (ε = 15.3 mM^-1^·cm^-1^ at 553 nm), and NAD(P)^+^ (ε = 6.2 mM^-1^·cm^-1^ at 340 nm) were used. Potential opine oxidase activity was estimated by measuring the production of H_2_O_2_ using the 4-aminoantipyrine peroxidase system [26]. The kinetic parameters, *K*_m_ and *k*_cat_ values, were calculated by a Lineweaver-Burk plot.

### Zymogram staining analysis

The purified enzyme was separated on non-denaturing PAGE with an 8% gel at 4°C. The gel was then soaked in 2 ml of staining solution consisting of 50 mM Tris-HCl (pH 9.0), 10 mM nopaline or octopine, 0.25 mM INT or NBT, and 0.06 mM PMS at room temperature for 15 min. Dehydrogenase activity appeared as a red (for INT) or violet band (for NBT).

### Determination of flavin cofactor

Cofactors in the purified enzyme were released by heat denaturation. After removal of the precipitate formed by centrifugation, the supernatant was used to identify the flavin cofactor(s) by HPLC. A HPLC analysis was conducted using a TSKgel ODS-80Tm column (4.6 × 150 mm, TOSOH). An isocratic elution (10 min) with 10 mM potassium phosphate, pH 6.0 followed by a linear gradient (30 min) between 0 and 70% methanol in the same solution was used for the elution. The flow rate was 1.0 ml/min, and elution was monitored by absorbance at 260 nm. Iron concentrations were determined using a Metallo Assay kit (AKJ Global Technology, Chiba, Japan).

### Amino acid sequence alignment and phylogenetic analysis

Protein sequences were analyzed using the Protein-BLAST and Clustal W program distributed by DDBJ (DNA Data Bank of Japan) (www.ddbj.nig.ac.jp). The phylogenetic tree was produced using the TreeView 1.6.1. program.

## Results and Discussion

### Gene cluster related to opine metabolism by *P*. *putida*

A protein blast analysis revealed that the homologous *AtOoxB-OoxA* and *AtNoxB-NoxA* genes existed as a gene cluster together with putative ABC-type transporter gene(s) on the genomes and/or plasmids of many bacteria; however, the mechanisms underlying opine catabolism were unclear. Although difficulties have been associated with expressing AtOoxAB and AtNoxAB in *E*. *coli* cells (see “Introduction”) [12, 13], we previously developed an expression system of recombinant proteins in *P*. *putida* cells (see below) [24, 25]. Therefore, we selected PP_4457 and PP_4456 (genes) from *P*. *putida* KT2440 as target genes, which corresponded to AtOoxB (39.6% of identity) and AtNoxB (40.4%), and AtOoxA (40.5%) and AtNoxA (48.1%), respectively (referred to as PpOdhB and PpOdhA, respectively) (Fig 1B). Furthermore, PP_4452 (gene) in the flanking region was similar to known NAD(P)H-dependent opine synthases, indicating the potential role of this gene cluster in opine metabolism. PpOdhB (AtOoxB and AtNoxB) and PpOdhA (AtOoxA and AtOoxA) were previously shown to be homologous to the β- and α-subunits of D-HypDH, respectively [14]. Based on this finding, we identified a provisional open-reading frame homologous to the γ-subunit of D-HypDH (referred to as PpOdhC) (Fig 1B). The 3’ part of *PpOdhB* and *PpOdhA* was overlapped by the 5’ part of *PpOdhC* and *PpOdhA*, respectively (Fig 2A), suggesting that *PpOdhB*-*C*-*A* (also *AtOoxB-* *C* *-A* and *AtNoxB-* *C* *-A*) encoded each subunit in the heteromeric structure.

### Expression of the recombinant protein in *P*. *putida* cells

The *P*. *putida* KT2442-oxyR1 strain has been shown to constitutively produce a soluble AhpC protein with a molecular mass of 24 kDa, a small subunit of alkyl hydroperoxide reductase [25]. Therefore, the *PpOdhB*-*C*-*A* operon, in which the (His)_6_-tag sequence was attached at the N-terminal of the *OdhB* gene, was expressed in the *ahpC* promoter in the cells of this *P*. *putida* strain (Fig 2A). The recombinant protein was successfully purified to homogeneity using immobilized metal (Ni^2+^) affinity chromatography and a buffer system containing Tween-20 (see the Materials and methods section) (Fig 2B), indicating tight binding to the cytoplasmic membrane, as described in preliminary studies [12, 13]. Inactivation was not detected after 1 months at –35°C.

Three major distinct bands with molecular masses of 46, 42, and 9 kDa, respectively, were observed in the SDS-PAGE analysis, and the N-terminal amino acid sequences of each protein were S-R-T-F-D-V-V-I-V-G, P-I-V-E-H-H, and M-D-V-S-I-F-K-K-L-P, which completely corresponded to those of PpOdhA, PpOdhB, and PpOdhC, respectively (GTG was used as a start codon for the *PpOdhA* gene) (referred to as α-, β-, and γ-subunits, respectively) (Fig 2A and 2B). The molar ratio of α:β:γ was ~1:1:1, as determined by image scanning of the SDS-PAGE peak areas using NIH ImageJ Ver. 1.45s software. The native molecular mass, estimated by gel filtration, was approximately 360 kDa, indicating that this protein molecule may be composed of a heterododecameric structure of subunits, i.e., α_4_β_4_γ_4_. The purified protein was orange-brown (inset of Fig 2C), indicating that any chromophore(s) must be bound to the protein (see below).

### PpOdhABC was identified as a novel flavin-containing opine dehydrogenase

In preliminary studies [12, 13], the (unpurified) AtNox and AtOox proteins were assayed by a quantitative determination of the urea liberated by arginase from L-arginine, which was produced by the oxidative cleavage of opines. On the other hand, PpOdhABC was sequentially similar to the so-called “dye-linked dehydrogenase”, which utilizes artificial electron acceptors such as 2,6-dichloroindophenol (Cl2Ind) instead of natural acceptors (see S2 Table) [14, 27]. Therefore, we attempted to assay (potential) opine oxidase activity in PpOdhABC using this system, and detected 27.9 unit/mg protein of specific activity toward nopaline (Table 1). The optimum pH for activity using Tris-HCl buffer was 9.0 (S1A Fig).

**Table 1:** Kinetic parameters for PpOpnDH.

Substrates	Electron acceptors	Specific activity ^a^ (units/mg protein)	*K*_m_	*k*_cat_	*k*_cat_/*K*_m_
			(mM)	(min^-1^)	(min^-1^·mM^-1^)
Nopaline	Cl2Ind	27.9±1.3	0.476±0.047	18900±1700	39800±370
Nopaline	PMS/INT	6.45±0.52	0.127±0.007	5490±320	43100±700
Nopaline	PMS/NBT	2.11±0.03	0.0772±0.0041	1390±40	18100±440
Nopaline	Ferricyanide	7.29±0.42	0.379±0.051	1040±47	2750±250
Nopaline	Cytochrome *c*	18.5±0.24	0.379±0.063	17920±2800	47300±380
Octopine	Cl2Ind	0.00780±0.00074	39.0±15.6	138±49	3.60±0.27

^a^ Under standard assay conditions described in the Materials and Methods section.

**Fig 3:**
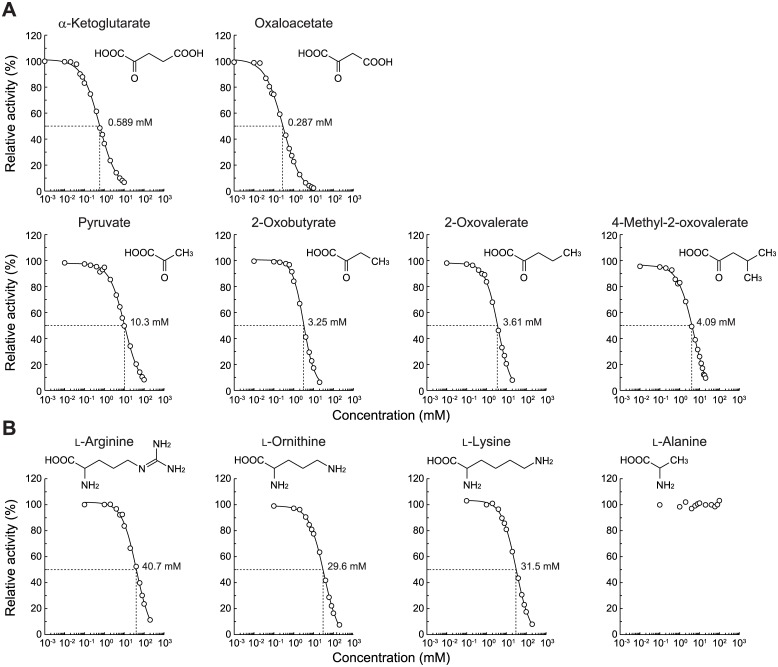
Inhibition study by α-keto acids (A) and L-amino acids (B) of PpOpnDH. Nopaline (0.1 mM) was used as a substrate. Relative specific activity values were expressed as percentages of the values obtained in the absence of an inhibitor. IC_50_ values were calculated by curve fitting using ImageJ software (http://rsb.info.nih.gov/ij/).

The catalytic efficiency (*k*_cat_/*K*_m_) value with nopaline (39,800 min^-1^·mM^-1^) was 11,000-fold lower than that with octopine (3.60 min^-1^·mM^-1^), and was attributed to 82-fold higher *K*_m_ and 137-fold lower *k*_cat_ values (Table 1). Since other opines were not available commercially, we alternatively estimated more detailed substrate specificity in an inhibition study (Fig 3). The IC_50_ value for α-ketoglutarate (0.589 mM) was ~17-fold higher than that for pyruvate (10.3 mM), thereby confirming the preference for nopaline over octopine, as described above. Furthermore, the inhibition of activity by α-ketoglutarate and oxaloacetate (with a carboxyl group) was 6.1- and 11-fold higher than that by 2-oxovalerate and 2-oxobutyrate (with methyl group), respectively. On the other hand, among several L-amino acids, significant inhibition was only observed in basic L-arginine, L-ornithine, and L-lysine. Their (similar) IC_50_ values were approximately two orders of magnitude lower than that of α-ketoglutarate. These results indicated that the enzyme recognized the *N*-substituted glutamic acid moiety of nopaline, and that nopalinic acid (ornaline), also found in crown gall tumor tissues [5], may also be the active substrate (Fig 1A).

In addition to Cl2Ind, other compounds were tested as electron acceptors. Significant activity was observed with PMS/INT, PMS/NTB, ferricyanide, and horse heart cytochrome *c* (but not NAD(P)^+^ or oxygen) (23, 7.6, 26, and 66% more specific activity than that of Cl2Ind); the enzyme utilized markedly broader artificial electron acceptors than other dye-linked dehydrogenases belonging to the same protein family (S2 Table). Of these, the *k*_cat_/*K*_m_ values of PMS/INT and horse heart cytochrome *c* were similar to those of Cl2Ind (Table 1). Substrate and electron acceptor specificities were also observed in the in-gel assay (zymogram staining) (Fig 2D). These results indicated that PpOdhABC was a dye-linked opine dehydrogenase (but not “oxidase”), and differed from known NAD(P)^+^-dependent enzyme(s) (referred to as PpOpnDH).

### Prosthetic group(s) in PpOpnDH

The spectrum of PpOpnDH showed the characteristics of a typical flavoprotein (maxima at approximately 350 and 450 nm) (Fig 2C) and the flavin compounds were identified as FAD and FMN. Alternatively, the cloning of *OdhA*, *OdhB*, and *OdhC* genes into different plasmid vectors for *E*. *coli* enabled each subunit of heteromeric PpOpnDH to be functionally characterized (the *E*. *coli* system was only used for this purpose because of the significantly lower expression level than that of the *P*. *putida* system, as described above) (Fig 2E). Only FAD was extracted from (almost) inactive OdhA, OdhB, OdhAB (co-expression of OdhA+B), OdhBC, and OdhAC, while FAD and FMN were only extracted from active OdhABC; the molar ratio of FAD:FMN was 1.9:1.0 (Fig 2F). Furthermore, iron contents per mole of OdhABC were ~7.5. Therefore, we concluded that PpOpnDH (α_4_β_4_γ_4_) contained 2 FAD (α- and β-subunits), 1 FMN (between the α- and β-subunits), and 1 [2Fe-2S] iron-sulfur cluster (γ-subunit) within the structural unit of αβγ, similar to D-HypDH (S2 Table) [14, 15]. On the other hand, the β-subunit of D-HypDH by itself completely functioned as a catalytic subunit, and the γ-subunit had no effect on the binding of FMN [15]. The former may have been due to the binding site(s) of nopaline being located not only within the β-subunit of PpOpnDH, but also other subunit(s), because of the markedly larger substrate than D-hydroxyproline. PpOdhAB exhibited low levels of activity, in spite of the absence of FMN (Fig 2E); the β- and α-subunit were both necessary for substrate binding.

### Characterization of OpnDH from *B*. *japonicum*

A further protein blast analysis revealed that the homologous gene cluster of *B*. *japonicum* with *PpOdhB-C-A* contained an additional *OdhB* gene, in the order of *BjOdhB*_*1*_ *-C-A-B*_*2*_ (Fig 1B). On the other hand, in the case of *B*. *thailandensis*, the order of the gene cluster was different from *P*. *putida*: *BtOdhC-A-B*. BjOdhB_1_ and BjOdhB_2_ were more similar to PpOdhB and BtOdhB, respectively.

**Fig 4:**
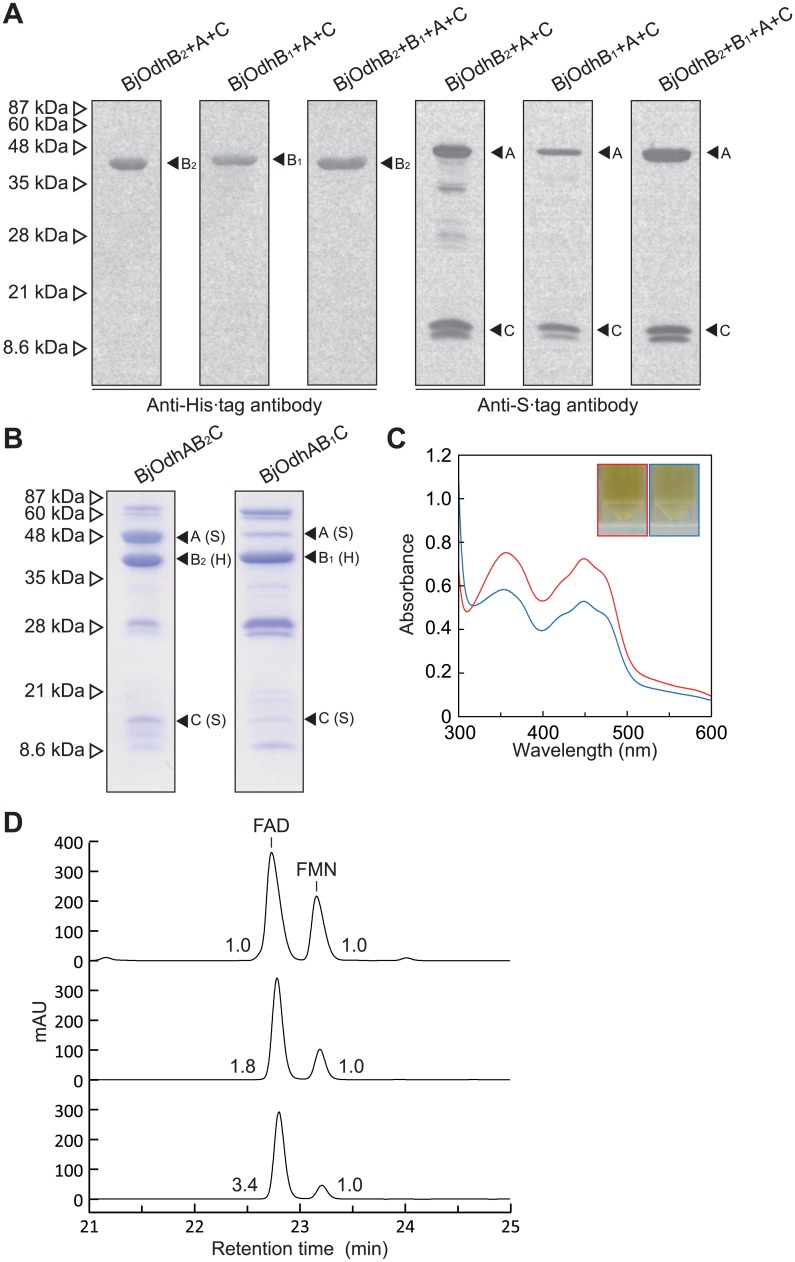
Characterization of opine dehydrogenases from *B*. *japonicum*. (A) A Western blotting analysis. BjOdhB_2_+A+C, BjOdhB_1_+A+C, and BjOdhB_2_+B_1_+A+C proteins were overexpressed in *E*. *coli* cells, and purified by a Ni-NTA column using the (His)_6_-tag attached to BjOdhB_2_ (for BjOdhB_2_+A+C and BjOdhB_2_+B_1_+A+C) or BjOdhB_1_ (for BjOdhB_1_+A+C), whereas S-tag was attached to other *Odh* genes. After SDS-PAGE of 20 μg protein per lane (Fig 4B), antibodies against the N-terminal (His)_6_-tag (left panel) and C-terminal S-tag (light panel) were used for immunoblotting. (B) An SDS-PAGE analysis of purified recombinant BjOdhAB_2_C and BjOdhAB_1_C. (C) Absorption spectra using 10 mg/ml BjOdhAB_2_C (red) and BjOdhAB_1_C (blue) solution (inset). (D) A HPLC analysis of prosthetic groups. Elution profiles of the standard mixture (the same as Fig 2F; upper), and extracts of BjOdhAB_2_C (middle) and BjOdhAB_1_C (lower). Numbers with peaks are the molar ratio of FAD:FMN.

In order to estimate the subunit assembly of the BjOdh protein, (His)_6_-tagged BjOdhB_1_ or BjOdhB_2_ was co-expressed together with S-tagged BjOdhA and BjOdhC in *E*. *coli* cells, and purified using Ni^2+^-affinity chromatography (Fig 4A and 4B). A western blotting analysis using the anti (His)_6_-tag and S-tag antibodies revealed that the purified proteins both contained not only each OdhB, but also OdhA and OdhC (referred to as BjOdhAB_1_C and BjOdhAB_2_C, respectively). Furthermore, their spectra were similar to that of PpOpnDH, and FAD and FMN were extracted from (orange-brown) them (Fig 4C and 4D). On the other hand, when (His)_6_-tagged BjOdhB_2_ was co-expressed together with BjOdhB_1_, BjOdhA, and BjOdhC, the purified protein did not contain BjOdhB_1_; the structure did not exist as BjOdhAB_1_B_2_C. Most of the purified BjOdhAB_2_C existed as α_4_β_4_γ_4_ (by gel-filtration), while BjOdhB_1_ was clearly dominant in purified BjOdhAB_1_C, thereby confirming that the molar ratio of FAD:FMN of the former (1.8:1.0) was similar to that of PpOdhABC.

Significant dehydrogenase activity with octopine (but not nopaline), using standard assay conditions, was detected in BjOdhAB_2_C: 0.504 unit/mg protein of specific activity (Table 2). In spite of the high specificity toward octopine, no significant difference was observed in the IC_50_ values between pyruvate and α-ketoglutarate: 54.0 and 26.1 mM, respectively (data not shown). On the other hand, since not only L-arginine, but also L-ornithine and L-lysine inhibited activity, octopinic acid and/or lysopine, also found in crown gall tumor tissues [2], may be the active substrate(s) (Fig 1A). The *k*_cat_/*K*_m_ value for octopine (using Cl2Ind; 224 min^-1^·mM^-1^) was 178-fold lower than that for nopaline of PpOpnDH, and this was mainly caused by a 40-fold higher *K*_m_ (19.5 mM). When large amounts of purified BjOdhAB_1_C were used, we detected poor dehydrogenase activity toward octopine (Table 3). This may have been caused by the small amount of the active enzyme (α_4_β_4_γ_4_) in the purified protein, as described above, confirming that PpOdhB alone was unable to function as a catalytic subunit (Fig 2E). The electron acceptor specificities (S2 Table) and optimum pH of BjOdhAB_2_C and BjOdhAB_1_C (S1B and S1C Fig) were similar to those of PpOpnDH. These results suggested that BjOdhAB_2_C and BjOdhAB_1_C both functioned as octopine-specific OpnDH, and differed from PpOpnDH (referred to as BjOpnDH_2_ and BjOpnDH_1_, respectively). A potential physiological role in opine catabolism may be limited to (active) BjOpnDH_2_ because the OdhA and OdhC proteins preferentially associated with OdhB_2_ over OdhB_1_.

**Table 2:** Kinetic parameters for BjOpnDH_2_.

Substrates	Electron acceptors	Specific activity ^a^ (units/mg protein)	*K*_m_	*k*_cat_	*k*_cat_/*K*_m_
			(mM)	(min^-1^)	(min^-1^·mM^-1^)
Octopine	Cl2Ind	0.504±0.047	19.5±4.4	4370±945	224±2
Octopine	PMS/INT	0.555±0.035	5.30±0.35	1490±91	281±2
Octopine	PMS/NBT	0.243±0.015	3.99±0.75	533±96	134±1
Octopine	Ferricyanide	0.889±0.052	11.8±1.0	4730±406	401±1
Octopine	Cytochrome *c*	0.919±0.041	3.63±0.06	1740±14	479±4
Nopaline	Cl2Ind	0.001>	N.D. ^b^	N.D.	N.D.

^a^ Under standard assay conditions described in the Materials and Methods section.

^b^ Not determined due to trace activity.

**Table 3:** Kinetic parameters for BjOpnDH_1_.

Substrates	Electron acceptors	Specific activity ^a^ (units/mg protein)	*K*_m_	*k*_cat_	*k*_cat_/*K*_m_
			(mM)	(min^-1^)	(min^-1^·mM^-1^)
Octopine	Cl2Ind	0.00953±0.00094	4.81±0.24	5.89±0.13	1.22±0.03
Octopine	PMS/INT	0.0132±0.0015	12.9±6.1	12.6±4.0	1.01±0.17
Octopine	PMS/NBT	0.0116±0.0024	1.29±0.14	5.56±0.10	4.33±0.39
Octopine	Ferricyanide	0.0241±0.0032	6.52±1.46	15.1±3.5	2.32±0.41
Octopine	Cytochrome *c*	0.0157±0.0024	3.54±0.33	10.4±2.4	2.94±0.25
Nopaline	Cl2Ind	0.001>	N.D. ^b^	N.D.	N.D.

^a^ Under standard assay conditions described in the Materials and Methods section.

^b^ Not determined due to trace activity.

### Phylogenetic analysis and molecular evolution

**Fig 5:**
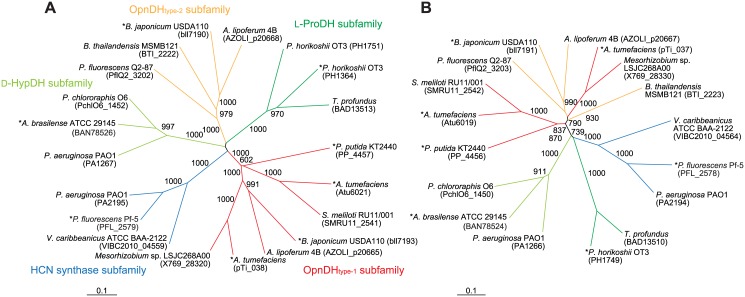
Phylogenetic analysis using β- (A) and α-subunits (B) of flavin-containing OpnDH. Letters in parentheses are the GenBank^TM^ accession numbers. The number on each branch indicates the bootstrap value. Proteins with asterisks were used for Fig 6.

**Fig 6:**
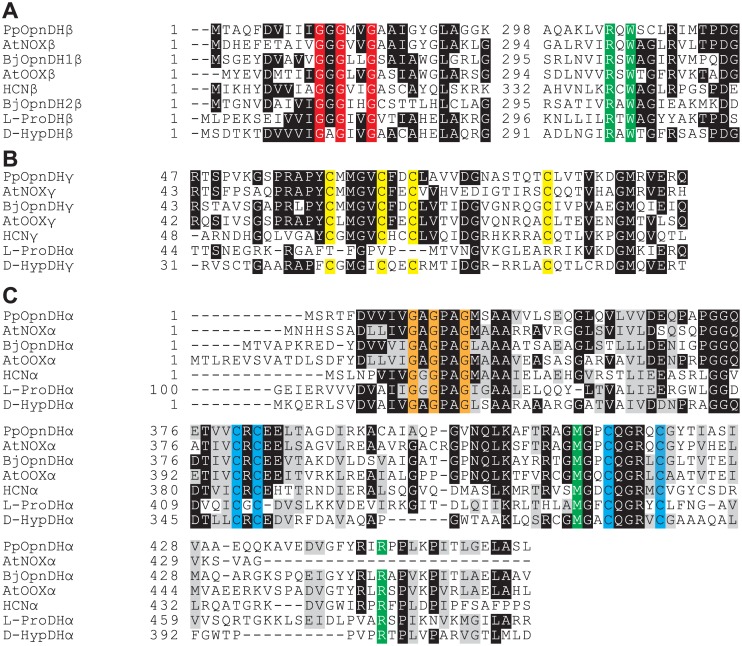
Partial multiple sequence alignment of deduced amino acid sequences of β- (A), γ- (B), and α-subunits (C) of flavin-containing OpnDH. White letters in black boxes indicate highly conserved amino acid residues. N-terminal ~120 amino acid residues of the α-subunit of L-ProDH corresponding to the γ-subunit of flavin-containing OpnDH and D-HypDH. Two ADP-binding motifs (Gly-*X*-Gly-*X*_2_-Gly) and [2Fe-2S]-binding motifs (Cys-*X*_4_-Cys-*X*_2_-Cys-*X*_11~12_-Cys and Cys-*X*-Cys-*X*_15~20_-Cys-*X*_4_-Cys) are shaded in red, orange, yellow, and cyan, respectively. FMN-binding sites are shaded in green.

Flavin-containing OpnDH (the β-subunit), which was the focus of this study, belongs to the D-amino acid oxidase (DAD) superfamily (pfam01266) [28], and differed from the known NAD(P)^+^-dependent enzyme (pfam02317). Although the DAD superfamily contains several oxidases for D-amino acid and sarcosine, a relatively high homology with OpnDH was found in hydrogen cyanide (HCN) synthase (EC 1.4.99.5) from bacteria [29], D-HypDH from bacteria [14, 15], and L-proline dehydrogenase (L-ProDH) from archaea (EC 1.5.5.2) [30, 31] with a heterooligomeric structure (Fig 5A and S2 Table). Although the subunit assembly of OpnDH (α_4_β_4_γ_4_) was the same as that of D-HypDH and HCN synthase, their sequential homologies were not high, suggesting the acquisition of substrate specificity after segmentation. PpOpnDH and BjOpnDH_1_ formed a single subfamily together with AtNox and AtOox (referred to as the OpnDH_type-1_ subfamily). Interestingly, BjOpnDH_2_ (and BtOpnDH) was not closely related to any of the subclasses including OpnDH_type-1_ (referred to as OpnDH_type-2_ subfamily), while a Protein-BLAST analysis revealed that this subfamily was close to the archaeal L-ProDH subfamily; however, L-proline was an inactive substrate for BjOpnDH_2_ (data not shown). Several specific motifs (amino acid residues), previously proposed in D-HypDH and L-ProDH [14, 30, 31], were significantly conserved in the primary structure(s) of OpnDH (Fig 6): two ADP-binding motifs at the N-terminus of the β- and α-subunits as (putative) the binding sites of 2 FAD; two different types of the [2Fe-2S]-binding motif in the γ- and α-subunits; (putative) FMN-binding sites in the α-subunit (and β-subunit; see below).

One of interesting results of the present study was that BjOdhB_1_ and BjOdhB_2_ both assembled with common α- and γ-subunits, and functioned as the same octopine dehydrogenase, thereby providing clear evidence for the acquisition of novel functions by “subunit-exchange”. Both possessed a motif of Arg-*X*-Trp for (putative) the binding sites of FMN (shaded in green) (Fig 6A), as well as D-HypDH and L-ProDH (see S2 Table). Therefore, an ancestor of this protein family may inherently possess FMN between the α- and β-subunits, and OpnDH_type-1_ and OpnDH_type-2_ may have acquired the same substrate specificity independently (convergent evolution). On the other hand, in the phylogenetic tree based on the α-subunits (Fig 6B), all OdhA proteins formed a single subfamily, suggesting another possibility that, for example, OpnDH_type-2_ had appeared by the acquisition of another *OdhB*_*2*_ gene into OpnDH_type-1_ (such as *B*. *japonicum*) with the subsequent loss of the *OdhB*_*1*_ gene. Another homomeric type of D-HypDH [14] and L-ProDH [27] has been identified that is not generally related to the β-subunit of the heteromeric enzymes; the discovery of such an OpnDH may strengthen this hypothesis.

### Physiological role of OpnDH

The degradation of toxic organic compounds by some *P*. *putida* strains has been extensively examined [32]. On the other hand, this bacteria, in addition to *B*. *japonicum*, also colonizes the rhizosphere of agronomically relevant plants at high population densities [33]; the origin of opines may be from rotting plants and/or plant exudates rather than biosynthesis. In case of endophytes, opines may be also provided without their being exuded. Tremblay *et al*. [17] reported that a *P*. *putida* strain isolated from a commercial nursery catabolized nopaline, thereby conforming to the substrate specificity of PpOpnDH. In contrast to *Agrobacterium* species, PpOpnDH and BjOpnDH genes are located on the chromosome. Indeed, large numbers of *Bradyrhizobium* species possess the homologous genes, whereas all the other *P*. *putida* strains, except for KT2440, do not. These findings suggest that *P*. *putida* KT2440 very recently acquired this ability by horizontal gene transfer (not plasmid transfer).

Marine invertebrates also possess NAD(P)^+^-dependent OpnDHs, which are classified into two groups: a mollusk/annelid type, enzymes for octopine, alanopine, and β-alanopine; a marine sponge type, enzymes for strombine and tauropine [8]. In spite of having the same substrate specificity, there was no sequence similarity between octopine dehydrogenases from marine invertebrates and bacteria, indicating that the convergent evolution of OpnDH occurred not only between flavin-containing and NAD(P)^+^-dependent enzymes, but also within each protein family. On the other hand, marine sponge type enzymes belong to the same protein superfamily (pfam02423) as ornithine cyclodeaminase (Fig 1) and Δ^1^-pyrroline-2-carboxylate (Pyr2C) reductase (EC 1.5.1.1): the latter catalyzes the NAD(P)H-dependent reduction of Pyr2C to L-proline, and is involved in *trans*-3-hydroxy-L-proline metabolism [34]. Therefore, phylogenetic relationships between flavin-containing OpnDH, D-HypDH, and L-ProDH may be physiologically linked to (ancestral) metabolic networks among opine(s), L-proline, L-arginine, and L-hydroxyproline.

## Supporting Information

S1 Fig Effects of pH on activities of PpOpnDH (A), BjOpnDH_2_ (B) and BjOpnDH_1_ (C). The assay was carried out with 50 mM potassium phosphate (pH7-8.5) (triangle), Tris-HCl (pH7.5–9) (circle), or 50 mM glycine-NaOH buffer (pH9-10) (square) instead of 50 mM Tris-HCl (pH9.0) under standard assay conditions described in the Materials and Methods section.
(EPS)

S1 Table Primers used in this study. ^a^ Lower case letters indicate additional bases for introducing the digestion sites of restriction enzymes in parentheses. ^b^ Only sense primers are shown. Underlining indicates mutated regions.
(DOCX)

S2 Table Comparison among flavin-containing OpnDH, D-HypDH, and L-ProDH. ^a^ N-terminal ~120 amino acid residues of the α-subunit corresponding to the γ-subunit of OpnDH and D-HypDH. ^b^ Four cysteine cluster. ^c^ Prosthetic group(s) in the same color may be structurally equivalent. Colors correspond to Fig 6. ^d^ The δ-subunit was sequentially similar to C-terminal ~90 amino acid residues of the α-subunit of L-ProDH from *P*. *horikoshii*. ^e^ Between α- and β-subunits. ^f^ Relative values (%) of specific activity (Tables 1–3).
(DOCX)
