# Supplementary material for: Characterization of Flavin-Containing Opine Dehydrogenase from Bacteria
Source: PLoS One. 2015 Sep 18;10(9):e0138434. doi: 10.1371/journal.pone.0138434 (PMC4575208; doi:10.1371/journal.pone.0138434)
Supplement: S1 Table — a Lower case letters indicate additional bases for introducing the digestion sites of restriction enzymes in parentheses. b Only sense primers are shown. Underlining indicates mutated regions. (DOCX) [file pone.0138434.s002.docx]

**Table S1. Primers used in this study**

| Primers ^a^ |  | Sequences ^a^ |
| --- | --- | --- |
| Cloning of the *PpOdhB* gene in pACYCDuet-1 | | |
| P1 (BglII) |  | 5’-catagatctgACTGCCCAATTTGATGTGATCATC-3’ |
| P2 (HindIII) |  | 5’-attaagctTCAGGAAGTTTTTTGAAGATCGAAAC-3’ |
| Cloning of the *PpOdhA* gene in pETDuet-1 | | |
| P3 (EcoRI) |  | 5’-catgaattcgCGAACGTTTGATGTGGTTATCGTCG-3’ |
| P4 (HindIII) |  | 5’-attaagcttCTATTTCTCCTCCACATCAAGTGAG-3’ |
| Cloning of the *PpOdhC* gene in pCOLADuet-1 | | |
| P5 (BamHI) |  | 5’-catggatccgGACGTTTCGATCTTCAAAAAACTTCC-3’ |
| P6 (HindIII) |  | 5’-attaagctTCACGACACTACACTCCGGCGGCC-3’ |
| Disruption of the BglII site in the *PpOdhB*-*PpOdhC*-*PpOdhA* fragment | | |
| P7 |  | 5’-GTGCCTCGTCCCcGATCTTGATGAG-3’ |
| P8 |  | 5’-CTCATCAAGATCgGGGACGAGGCAC-3’ |
| Cloning for (His)_6_-*PpOdhABC* gene in pUCP26KmAhpC_p_ | | |
| P9 (XhoI) |  | 5’-ccatctcgagCACCATCACCATCACCATggatcc-3’ |
| P10 (EcoRI) |  | 5’-gcttgaattcTCACCAATAAAAAACGCCCGGC-3’ |
| Cloning of the *BjOdhB_2_* gene in pACYCDuet-1 | | |
| P11 (BamHI) |  | 5’-catggatccgACTGGAAACGTGGATGCGATCGTCATTGG-3’ |
| P12 (HindIII) |  | 5’-attaagcttCTAGAGCGTGGAAGGGTGGAAACGGTCG-3’ |
| Cloning of the *BjOdhB_1_* gene in pACYCDuet-1 | | |
| P13 (BamHI) |  | 5’-catggatccgTCAGGAGAATACGACGTCGCCGTCGTCGG-3’ |
| P14 (HindIII) |  | 5’-attaagcttCTAGTAGCCGCTGTTGTTGGCCGCGCC-3’ |
| Cloning of the *BjOdhA* gene in pETDuet-1 | | |
| P15 (NdeI) |  | 5’-cgaccatATGACTGTGGCTCCCAAGCGCGAAGATTAC-3’ |
| P16 (XhoI) |  | 5’-attctcgagTCCGCGCACCACGGCCTTGACGTCG-3’ |
| Cloning of the *BjOdhC* gene in pCOLADuet-1 | | |
| P17 (NdeI) |  | 5’-cgaccatATGTTTAGACGATCCGAACAGGACAAAC-3’ |
| P18 (XhoI) |  | 5’-attctcgagTCTTCCGATCTCCCGCTTGCCCTTC-3’ |
| Cloning of the *BjOdhB_1_* and *BjOdhB_2_* genes in pACYCDuet-1 | | |
| P19 (NdeI) |  | 5’-gaccatATGTCAGGAGAATACGACGTCGCCGTCGTCGG-3’ |
| P20 (SalI) |  | 5’-attgtcgacGTAGCCGCTGTTGTTGGCCGCGCC-3’ |
