# Supplementary material for: Characterization of Flavin-Containing Opine Dehydrogenase from Bacteria
Source: PLoS One. 2015 Sep 18;10(9):e0138434. doi: 10.1371/journal.pone.0138434 (PMC4575208; doi:10.1371/journal.pone.0138434)
Supplement: S2 Table — a N-terminal ~120 amino acid residues of the α-subunit corresponding to the γ-subunit of OpnDH and D-HypDH. b Four cysteine cluster. c Prosthetic group(s) in the same color may be structurally equivalent. Colors correspond to Fig 6. d The δ-subunit was sequentially similar to C-terminal ~90 amino acid residues of the α-subunit of L-ProDH from P. horikoshii. e Between α- and β-subunits. f Relative values (%) of specific activity (Tables 1–3). (DOCX) [file pone.0138434.s003.docx]

**Table S2. Comparison between flavin-containing OpnDH, D-HypDH, and L-ProDH**

| Microorganisms  (Enzymes) | | *P. putida*  (PpOpnDH) | *B. japonicum*  (BjOpnDH_2_) | *B. japonicum*  (BjOpnDH_1_) | *P. aeruginosa*  (D-HypDH) | *P. horikoshii*  (L-ProDH) ^a^ | *T. profundus*  (L-ProDH) |
| --- | --- | --- | --- | --- | --- | --- | --- |
| Domain | | Bacteria | Bacteria | Bacteria | Bacteria | Archaea | Archaea |
| Sublocation | | Membrane | Membrane | Membrane | Membrane | Cytosol | Cytosol |
| Subunits | | α_4_β_4_γ_4_ | α_4_β_4_γ_4_ | α_4_β_4_γ_4_ | α_4_β_4_γ_4_ | α_4_β_4_ | αβγδ |
| Prosthetic group(s) ^c^ | α | FAD  Fe-4Cys | FAD  Fe-4Cys | FAD  Fe-4Cys | FAD  Fe-4Cys | ATP  Fe-4Cys ^b^ | FAD  Fe-4Cys (δ) ^d^ |
|  | γ | 2Fe-2S | 2Fe-2S | 2Fe-2S | 2Fe-2S | − | 2Fe-2S |
|  | β | FAD | FAD | FAD | FAD | FAD | FAD |
|  | α/β ^e^ | FMN | FMN | FMN | FMN | FMN | − |
| Substrates | | Nopaline | Octopine | Octopine | D-Hyp | L-Proline | L-Proline |
| Electron acceptor | Cl2Ind | 100 ^f^ | 100 ^e^ | 100 ^e^ | 0 | 100 | 100 |
|  | Ferricyanide | 26 | 176 | 253 | 0 | 100 | 207 |
|  | PMS/INT | 23 | 110 | 139 | 100 | 0 | 51 |
|  | PMS/NBT | 7.6 | 48 | 122 | 7 | 0 | 24 |
|  | Cytochrome *c* | 66 | 182 | 165 | 0 | 0 | 0 |
|  | NAD^+^  NADP^+^ | 0 | 0 | 0 | 0 | 0 | 0 |
| References | | This study | This study | This study | [14] | [30] | [31] |
| ^a^ N-terminal ~120 amino acid residues of the α-subunit corresponding to the γ-subunits of OpnDH and D-HypDH. | | | | | | | |
| ^b^ Four cysteine cluster. | | | | | | | |
| ^c^ Prosthetic group(s) in the same color may be structurally equivalent. Colors correspond to Figure 6. | | | | | | | |
| ^d^ The δ-subunit was sequentially similar to the C-terminal ~90 amino acid residues of the α-subunit of L-ProDH from *P. horikoshii*. | | | | | | | |
| ^e^ Between α- and β-subunits. | | | | | | | |
| ^f^ Relative values (%) of specific activity (Tables 1-3). | | | | | | | |
